# Editorial: Clinical pharmacist service promotes the improvement of medical quality, volume II

**DOI:** 10.3389/fphar.2025.1714684

**Published:** 2025-10-14

**Authors:** Hao Li, Jianrong Zhang, Shusen Sun, Wei Luan, Zhi-Chun Gu

**Affiliations:** ^1^ Clinical Research Ward, Clinical Research Center, Shanghai Children’s Medical Center, Shanghai Jiao Tong University School of Medicine, Shanghai, China; ^2^ Victorian Cancer Registry, Cancer Council Victoria, Melbourne, Australia; ^3^ Department of Pharmacy Practice, College of Pharmacy and Health Sciences, Western New England University, Springfield, MA, United States; ^4^ Department of Nursing, Shuguang Hospital, Shanghai University of Traditional Chinese Medicine, Shanghai, China; ^5^ Department of Pharmacy, Ren Ji Hospital, Shanghai Jiao Tong University School of Medicine, Shanghai, China

**Keywords:** clinical pharmacist, medication therapy management, pharmaceuticalcare, pharmacovigilance, medical education and counseling

## Introduction

This Research Topic, “Clinical Pharmacist Service Promotes the Improvement of Medical Quality,” was launched in 2023, with the first volume including 27 articles (Li et al., 2024). We received 115 submissions for the current volume, of which 52 were accepted following peer review. This notable increase in submissions reflects the growing global recognition of clinical pharmacists’ contributions to improving healthcare quality.

The accepted publications in this volume originate from a wide range of countries, including but not limited to China, Ethiopia, Germany, Brazil, and Türkiye. The roles and integration of pharmacist services within the healthcare systems of these countries vary significantly. In settings with well-established clinical pharmacy services, pharmacists are often integral members of multidisciplinary teams, managing complex medication therapies (Muscat et al., 2024). In contrast, in resource-limited settings, the focus is more on ensuring fundamental appropriate medicine use and addressing urgent drug-related problems (Spinks et al., 2023). This contextual information is crucial for interpreting the diverse pharmacist activities described in the following sections.

The above conditions, in a way, reflect the breadth of the Research Topic addressed in the 52 publications. It is important to note that while some studies (e.g., those on medication therapy management, pharmaceutical care, and pharmacovigilance) fall within the formal responsibilities of hospital pharmacist services and represent the core theme of this Research Topic, other projects led by pharmacists (e.g., exploration of novel therapeutic regimens and drug targets, biomarker discovery) would not typically be considered part of the traditional formal hospital pharmacist service. This distinction will be further explored in the overall discussion at the end of this editorial.

A key development in this volume is the addition of articles addressing policy and cost-effectiveness analyses that were absent in the previous Research Topic. In contrast, only a limited number of submissions have focused on clinical pharmacists’ participation in clinical trials or their roles in education and training. These studies provide valuable insights that further support the rational use of medicines in clinical practice.

## Medication therapy management service and pharmaceutical care

Clinical pharmacists’ direct involvement in patient care continues to demonstrate a significant impact across diverse clinical settings. Multiple studies in this section highlight how pharmacist-led services optimize therapeutic outcomes.

Pharmacist interventions are central to improving the effectiveness of healthcare. In transplant medicine, Chen et al. conducted a retrospective cohort study showing that a pharmacist-led, closed-loop immunosuppressant service improved drug level management precision and enhanced renal function outcomes in kidney transplant recipients.

In a prospective study, Endalifer et al. reported that clinical pharmacists effectively identified and resolved drug-related problems (DRPs) in medical wards in a hospital of Ethiopia. Most pharmacist’s recommendations are accepted by prescribers, leading to positive clinical and economic outcomes.

Anticoagulation management remains a critical area of pharmacist involvement. In a retrospective study, Teka et al. found that warfarin anticoagulation quality was poor among outpatients in Western Ethiopia. Concurrent aspirin use and congestive heart failure were identified as the independent predictors of suboptimal control.

Integration of technology and advanced analytics into pharmaceutical care is another important theme. In a machine learning–based study, Li et al. demonstrated that a pharmacist-led surgical prescription optimization service significantly improved prescription appropriateness and key outcomes, including reduced hospital stays and costs. Qin et al. developed and validated machine learning models, showing that extreme gradient boosting effectively predicted cancer-associated disseminated intravascular coagulation and 30-day mortality in critically ill patients with colorectal cancer, outperforming established clinical scoring systems.

Personalized medical approaches have also advanced. Yuan et al. proposed integrating pharmacogenomics into clinical practice through an information software platform using colorectal cancer as an example. They suggested that this approach could enhance personalized medication management and expand pharmacists’ roles in patient care. In a retrospective cohort study by Chen et al., pharmacogenomics-guided treatment improved medication adherence and reduced antidepressant switching in patients with major depressive disorders.

Precision dosing strategies have been developed for psychiatric medications. Chen et al. created a population pharmacokinetic model that provided optimized initial dosing recommendations for quetiapine in patients with schizophrenia, with adjustments based on body weight and concurrent fluvoxamine or duloxetine use. In another study, Chen et al. proposed a model-informed precision dosing strategy for olanzapine in patients with bipolar disorder, recommending dose adjustments based on body weight and concurrent quetiapine use.

Risk prediction tools further illustrated pharmacists’ contributions. Han et al. developed and validated a nomogram that accurately predicted bleeding risk in cardiac surgery patients receiving combined anticoagulant and antiplatelet therapy, outperforming the existing scoring systems.

Critical-care pharmacy services have received substantial attention. Wei et al. proposed a practical operational model for critical care pharmacists in China, outlining workflows for medication evaluation, ward rounds, and follow-ups to optimize patient care and reduce drug-related problems. Özgan et al. conducted a prospective non-randomized controlled study and showed that clinical pharmacist interventions significantly reduced drug-related problems and improved nutritional outcomes in critically ill patients with renal dysfunction in Türkiye. The economic value of these services was confirmed by Wei et al. in a prospective cohort study, which demonstrated that pharmaceutical care for critically ill patients was cost-effective and significantly reduced medication errors, adverse drug events, intensive care unit (ICU) length of stay, and overall treatment costs.

The management of chronic diseases has also been addressed. Ma et al. conducted a retrospective multicenter cohort study and found that higher medication complexity in patients with COPD was associated with reduced follow-up attendance, highlighting the need for regimen simplification and enhanced patient support.

## Pharmacovigilance study

Medication safety monitoring and adverse event prevention are another major focus of this Research Topic, with studies employing diverse methodologies to strengthen pharmacovigilance.

Technology-enabled safety monitoring was assessed by Bauer et al., who conducted a clinical-pharmaceutical evaluation and found that while a clinical decision support system generated numerous alerts, most were not clinically relevant in a hospital in Germany. This demosntrates the essential role of clinical pharmacists in interpreting alerts and conducting comprehensive medication reviews to ensure patient safety. In a retrospective observational study, Albanese et al. showed that a trigger tool effectively identified adverse events in a neonatal ICU, with prematurity, low birth weight, and prolonged hospitalization identified as major risk factors in a hospital in Brazil.

Large-scale real-world studies have further advanced pharmacovigilance. Duan et al. conducted a multicenter study demonstrating that placental peptide injection had a very low adverse drug reaction rate (0.03%), supporting its favorable safety profile. Fang et al. performed a retrospective analysis showing that female sex, combination immunotherapy, and pre-existing autoimmune disease were significant predictors of both increased risk and earlier onset of immune-related adverse events in patients with cancer receiving immune checkpoint inhibitors.

The safety of anticoagulation in vulnerable populations has also been highlighted. Addisu et al. conducted a multicenter cross-sectional study and found that warfarin anticoagulation quality was suboptimal in older Ethiopian patients with atrial fibrillation in Ethiopia. Poor therapeutic control was associated with advanced age, chronic kidney disease, and infrequent monitoring, whereas bleeding events were linked to diabetes and higher stroke risk scores.

The comparative safety profiles of the therapeutic agents were clarified through a meta-analysis. In a systematic review and network meta-analysis, Chen et al. concluded that triazole antifungals, particularly isavuconazole, were more effective and safer than amphotericin B deoxycholate as first-line therapy for invasive *aspergillosis*. In a meta-analysis, Ren et al. found that, in Asian stroke patients, low-dose antiplatelet therapy reduced bleeding risk without compromising efficacy compared to standard doses. Fu et al. showed in a network meta-analysis that penpulimab plus chemotherapy was most effective for first-line advanced non-small cell lung cancer, although efficacy and safety varied according to PD-L1 expression and histology. In a systematic review and meta-analysis, Zhang et al. reported that a penicillin allergy label was associated with increased risks of mortality, acute heart failure, ICU admission, and mechanical ventilation. Qu et al. concluded in another systematic review and meta-analysis that delayed-release posaconazole provided more stable concentrations than oral suspension and was less affected by diarrhea or acid-suppressing agents.

Novel predictive approaches based on machine learning have also been reported. Hua et al. developed and validated models that effectively identified inpatients at risk of coagulation dysfunction associated with specific β-lactam antibiotics, demonstrating the utility of proactive surveillance. Hu et al. conducted a systematic review and meta-analysis, showing that machine learning models based on electronic health records could effectively predict adverse drug events.

Drug-specific safety concerns and interactions were further explored. Zheng et al. found that pre-existing hypertension and age >60 years were significant predictors of bevacizumab-induced hypertension in patients with metastatic cancer patients. Yang et al. reported that diltiazem significantly increased tacrolimus concentrations in pediatric nephrotic syndrome patients, with stronger interactions in CYP3A5 expressers. Similarly, Zong et al. demonstrated that calcium channel blockers increased tacrolimus levels in renal transplant recipients, with the CYP3A5 genotype influencing the magnitude of interaction, especially with amlodipine.

Finally, Xia et al. conducted a comparative study among lobular breast cancer patients and found that those with T3N0M0 stage had a higher risk of lung-related mortality than those with T2N1M0 stage, and radiotherapy was identified as a significant risk factor for this outcome.

## Participation in clinical trials

Clinical pharmacists are increasingly engaging in clinical trials, an emerging and vital area of practice. While the Good Clinical Practice guideline designates ultimate responsibility to the Principal Investigator, who is a clinician, and particular phase I studies demand specific medical expertise, the unique skills of clinical pharmacists are indispensable. They contribute significantly across the trial lifecycle. In the planning phase, their expertise is crucial in designing protocols, particularly in formulating dosing regimens and safety monitoring plans. During execution, they manage the integrity, storage, and dispensing of investigational products. Furthermore, they provide essential patient education on medication use and adherence counseling. In the evaluation phase, clinical pharmacists play a key role in the initial assessment and accurate documentation of adverse events, thereby enhancing the overall quality and safety of the clinical trial. However, it is noteworthy that only two manuscripts focusing on this important theme were included in the current Research Topic.

Xie et al. conducted a clinical study showing that remimazolam provided anesthetic efficacy equivalent to propofol in older patients undergoing hysteroscopic surgery but with a superior safety profile, including fewer incidences of respiratory depression, injection pain, and hypotension.

Zhang et al. carried out a phase I clinical trial of NH130, a potential treatment for Parkinson’s disease psychosis. The trial indicated a favorable pharmacokinetic and safety profile in healthy volunteers, consistent with predictions from a physiologically based pharmacokinetic model.

## Medical education and counseling

Enhancing pharmacy education and patient counseling are essential for advancing the profession. Guo et al. conducted a discrete choice experiment, showing that Chinese undergraduate pharmacy students strongly preferred case-based learning with scenario simulations and authentic cases led by clinical instructors. These attributes outweigh factors, such as group size and examination format.

In a cross-sectional study, Mekonnen et al. found that community pharmacy professionals in Northwest Ethiopia possessed good knowledge of substandard and falsified medicines, but their practical application of this knowledge was inadequate, highlighting the need for targeted education and training.

Patient adherence is another important theme. Dagnew et al. conducted a multicenter cross-sectional study in Northwest Ethiopia and reported that one-third of cardiovascular patients had low medication adherence, with older age, alcohol use, and polypharmacy identified as significant determinants.

Global trends were analyzed by Wu et al., who performed a bibliometric analysis of home pharmaceutical care. Their findings indicate that research in this field is predominantly led by developed countries, with current hotspots focused on polypharmacy and medication reconciliation. They also highlight the need for stronger international collaboration and greater research development in resource-limited settings.

## Indications of potential treatment targets

Several studies have explored novel therapeutic strategies and biomarkers for hepatocellular carcinoma. Luo et al. conducted a causal analysis integrating Mendelian randomization and colocalization, identifying circulating proteins such as matrix metalloproteinase-12 (MMP12) and acid sphingomyelinase (ASM) as potential therapeutic targets for chronic obstructive pulmonary disease (COPD**)**. They further suggested that the risk accelerated by these proteins could be modulated by lifestyle factors.

Cong et al. conducted an experimental study demonstrating that the neuroprotective effects of irisin against cerebral ischemia–reperfusion injury were mediated by sirtuin 3 (SIRT3) activation, as the benefits were diminished when SIRT3 was inhibited.

Wang et al. employed a multi-omics approach to develop a glycolysis-related risk-score model for esophageal squamous cell carcinoma. The model effectively stratified patients into prognostic groups and identified subtype-specific drug sensitivities, thereby offering a foundation for personalized treatment.

Liu et al. presented a case report showing that sulodexide was an effective and safe long-term anticoagulant in a complex case of myasthenia gravis with a giant thymoma complicated by heparin-induced thrombocytopenia and bleeding.

Biomarker discovery has also been advanced through epidemiological studies. Yu et al., using NHANES data (1999–2004), found that a higher hemoglobin-to-red blood cell distribution width ratio was associated with a lower prevalence of peripheral artery disease, suggesting a protective role. Similarly, Wang et al. analyzed NHANES data from 2001 to 2018 and reported an inverse association between this ratio and coronary artery disease risk, further supporting its protective potential. Zhang et al. conducted a cross-sectional analysis identifying waist circumference as the strongest predictor of hypertension, particularly in younger adults, whereas the predictive value of anthropometric indices diminished in older populations.

Therapy optimization studies further support clinical decision-making. In a Bayesian network meta-analysis, Chen et al. found that granisetron and oxycodone were the most effective interventions for preventing etomidate-induced myoclonus, although the certainty of evidence was moderate to low. Li et al. conducted a network meta-analysis, suggesting that sequential administration of androgen receptor signaling inhibitors after docetaxel chemotherapy was as effective as concomitant therapy for metastatic hormone-sensitive prostate cancer, providing a viable treatment alternative. Using Mendelian randomization, Zhu et al. identified circulating metabolites, such as acetate and linoleic acid, which are causally linked to breast cancer risk, highlighting the role of lipid metabolism in disease development.

## Policy and cost-effectiveness analysis

This section highlights the economic and policy dimensions of clinical pharmacy services. Fang et al. conducted a retrospective real-world study demonstrating that the implementation of a national health insurance coverage policy substantially increased the use of mecapegfilgrastim in cancer patients, although disparities persisted by patient location, cancer type, and tumor stage.

Yang et al. conducted a multicenter survey in Hebei Province, China, showing that inpatients had limited awareness of clinical pharmacy services. Nevertheless, most expressed a willingness to pay for such services, with health insurance reimbursement identified as the preferred payment method.

To address regulatory gaps, Zhao et al. developed an expert consensus providing a framework for the off-label use of drugs for rare hematologic diseases in China, bridging the gap between approved labeling and clinical needs.

Cost-effectiveness studies provide further insights into healthcare decision making. Lang et al. concluded that tislelizumab plus chemotherapy is a cost-effective first-line treatment for advanced gastric cancer in China, but not in the United States, based on willingness-to-pay thresholds in each setting. Cai et al. found that adding atezolizumab to bevacizumab and chemotherapy was not cost-effective for metastatic cervical cancer in China because the incremental cost exceeded the accepted thresholds. Zhang et al. conducted a network meta-analysis identifying vonoprazan-based triple therapy as the most effective regimen and high-dose dual therapy as the safest strategy for *Helicobacter pylori* eradication in East and Southeast Asian adult populations.

## Future perspectives

Most papers in this volume focus on pharmaceutical care and pharmacovigilance. In contrast, relatively few submissions examined clinical pharmacists’ participation in clinical trials. We hope that clinical pharmacists will play a more active role in clinical trials, particularly by conducting in-depth research on pharmacokinetics, drug safety, and optimal treatment timing, while also providing pharmaceutical care throughout the trial process (Li et al., 2012; Zhang et al., 2025).

It is also pertinent to consider the limitations observed in the studies published under this Research Topic. Many studies were single-center, had limited sample sizes, or were observational in nature, which may affect the generalizability of the findings and should be considered when assessing the role and limits of pharmacists in the clinical environment. This underscores the need for more rigorously designed, multi-center studies to provide higher levels of evidence.

We encourage the development of multicenter clinical trials led by clinical pharmacists to generate high-quality evidence demonstrating their role in improving healthcare quality and advancing models of care across the disease continuum (Martin et al., 2018; Chan et al., 2023; Holland et al., 2023; Villiet et al., 2025).

In addition, we were pleased to have received policy research papers on this Research Topic. Moving forward, we hope that clinical pharmacists will engage more actively in social surveys and pharmacoeconomic analyses using their findings to derive robust policy recommendations that can guide healthcare development (Chasseigne et al., 2020).
